# Machine Learning as a “Catalyst” for Advancements in Carbon Nanotube Research

**DOI:** 10.3390/nano14211688

**Published:** 2024-10-22

**Authors:** Guohai Chen, Dai-Ming Tang

**Affiliations:** 1 Nano Carbon Device Research Center, National Institute of Advanced Industrial Science and Technology (AIST), Tsukuba Central 5, 1-1-1 Higashi, Tsukuba 305-8565, Japan; 2 Research Center for Materials Nanoarchitectonics (MANA), National Institute for Materials Science (NIMS), Tsukuba 305-0044, Japan; 3 Institute of Pure and Applied Sciences, University of Tsukuba, Tsukuba 305-8571, Japan

**Keywords:** carbon nanotube, machine learning, data-driven, deep learning, artificial intelligence, chemical vapor deposition, catalyst, synthesis, characterization, application

## Abstract

The synthesis, characterization, and application of carbon nanotubes (CNTs) have long posed significant challenges due to the inherent multiple complexity nature involved in their production, processing, and analysis. Recent advancements in machine learning (ML) have provided researchers with novel and powerful tools to address these challenges. This review explores the role of ML in the field of CNT research, focusing on how ML has enhanced CNT research by (1) revolutionizing CNT synthesis through the optimization of complex multivariable systems, enabling autonomous synthesis systems, and reducing reliance on conventional trial-and-error approaches; (2) improving the accuracy and efficiency of CNT characterizations; and (3) accelerating the development of CNT applications across several fields such as electronics, composites, and biomedical fields. This review concludes by offering perspectives on the future potential of integrating ML further into CNT research, highlighting its role in driving the field forward.

## 1. Introduction

### 1.1. Machine Learning

Recently, machine learning (ML), a new paradigm, has demonstrated great potential as a tool for materials research [1,2,3]. ML is a subset of artificial intelligence (AI) concerned with the development and study of algorithms and statistical models that can learn from data, generalize to unseen data, and thus perform tasks without explicit instructions. Instead of following predetermined instructions, ML systems learn from data to recognize patterns, make decisions, and improve their performance over time [4,5,6,7,8,9,10].

**Figure 1:**
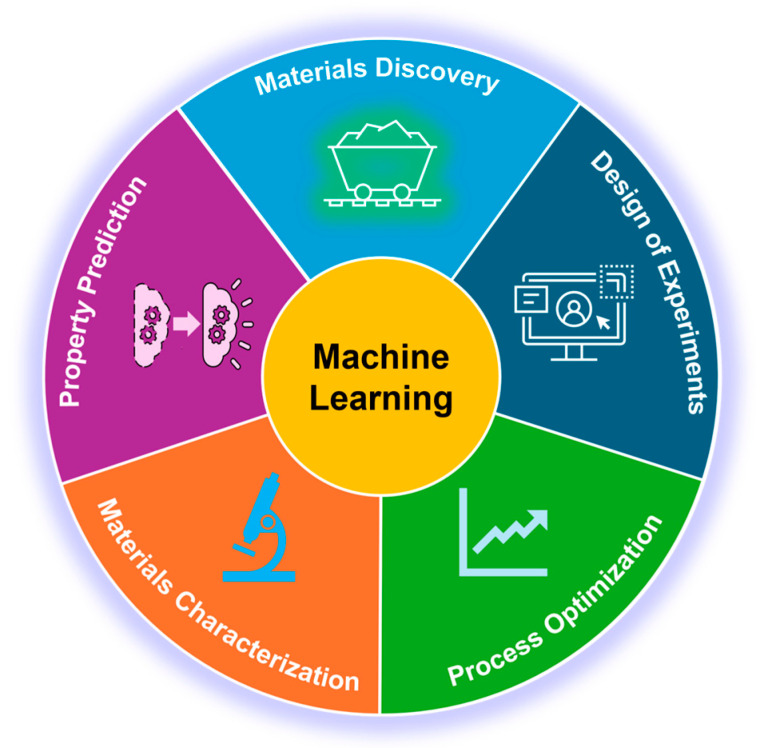
Machine learning-assisted materials research in various aspects.

ML has shown a wide range of applications across various domains, such as materials research, healthcare, finance, retail, autonomous vehicles, language processing, etc. Specifically, in the field of materials science, ML has been applied to various aspects and demonstrated great success [11]. Some of them are briefly discussed here (Figure 1).

(1) Materials discovery. ML has significantly accelerated the discovery of new materials, showing great advantages compared with traditional approaches, which have been slow and labor-intensive, involving extensive experimentation and trial-and-error processes [12,13]. ML offers a data-driven alternative, enabling the prediction of new materials with desired properties. With the assistance of ML, high-throughput screening allows thousands or even millions of potential compounds to be evaluated computationally using trained ML models before selecting a few candidates for real experimental testing. Additionally, inverse design becomes feasible, where researchers begin with the desired material properties and use ML algorithms to identify the structures or compositions that can achieve those properties. For instance, Rao et al. used an iterative scheme that combines ML, density functional theory, experiments, and thermodynamic calculation to find two new invar alloys with extremely low thermal expansion out of millions of candidates. The alloys are both compositionally complex, high-entropy materials, thus demonstrating the power of this approach for materials discovery [14].

(2) Process optimization. ML is also revolutionizing the optimization of manufacturing/synthesizing processes in materials science. These processes often involve multiple complex variables, such as temperature, pressure, gas, and time, which must be carefully controlled to achieve optimal results; however, the variable space is far too vast to exhaustively explore experimentally. ML enables more efficient optimization by analyzing complex datasets and identifying the most critical parameters. For example, in the field of chemical vapor deposition (CVD) synthesis of single-walled carbon nanotubes (SWCNTs), Rao et al. utilized ML to find optimal synthesis conditions within one hundred experiments to selectively grow SWCNTs within a narrow diameter range [15]. Lin et al. predicted growth conditions toward addressing the synthetic trade-off between the crystallinity and growth efficiency of SWCNT forests by training an ML regression model and validating it in less than 50 tests [16]. ML has significantly advanced process optimization, especially for complex multivariable systems.

(3) Materials characterization. ML is being integrated into the characterization of materials, where it can reveal hidden patterns in complex datasets and automate the analysis of experimental results [17,18]. ML, particularly deep learning techniques such as convolutional neural networks (CNNs), is used to analyze microscopy images to automatically classify microstructural features in the images, such as defects, phase boundaries, grain structures, particles, etc. ML is also used to analyze data from various spectroscopic techniques, including Raman [19,20], infrared [21,22], and X-ray photoelectron spectroscopy (XPS) [23,24,25,26]. ML can deconvolute complex spectra, identify chemical species, and quantify material composition. For example, Dee et al. performed quantitative analysis of catalyst nanoparticles for CNT synthesis using high-resolution, high-rate video capture of environmental transmission electron microscopy experimentation coupled with automated image processing, involving computer vision ML algorithms and CNNs [27]. Automation using ML accelerates the analysis process and provides more consistent and objective results [28].

(4) Property prediction. Predicting the properties of materials, such as mechanical, thermal, electronic, and optical properties, based on their composition and structure is one of the key applications of ML in materials science. This is particularly useful for materials where experimental property measurement is challenging or time-consuming. For instance, Hajilounezhad et al. predicted mechanical properties, including the stiffness and buckling properties of CNT forests, using an image-based ML classifier model, showing an accuracy of >91% [29].

(5) Design of experiments (DoE). ML greatly enhances the DoE by enabling more efficient exploration of the experimental parameter space. Traditionally, DoE involves systematically varying a few parameters to identify optimal conditions. However, this approach can be limited when dealing with high-dimensional spaces with many variables. Leveraging ML analysis alongside the DoE approach allows for using limited resources, particularly time, more efficiently and increases the likelihood of achieving a true optimum. For example, Cao et al. successfully optimized the materials and devices of organic photovoltaics via the combination of ML and DoE [30].

In summary, ML is profoundly impacting various aspects of materials science, from the discovery of new materials and the optimization of manufacturing/synthesizing processes to the prediction of material properties and the automation of materials characterization. These advancements are accelerating the pace of innovation, reducing the time and cost associated with materials development, and enabling the design of materials with unprecedented precision and desired functionality. As ML techniques continue to evolve, their integration into materials science will likely become even more pervasive, driving further breakthroughs and opening up new possibilities in this dynamic field.

### 1.2. Carbon Nanotubes

CNTs represent a sub-realm of materials science and a cross-discipline field that intersects with chemistry and applied physics. CNTs, a specific allotrope of carbon, are cylindrical structures characterized by diameters in the nanometer range and lengths that can span from nanometers to centimeters. Due to their unique one-directional structures, CNTs exhibit exceptional mechanical, electrical, and thermal properties. Specifically, CNTs possess remarkable tensile strength, excellent electrical conductivity, and superior thermal stability. These qualities make CNTs highly promising for a broad spectrum of applications, including electronics, composites, energy storage, and biomedical engineering. In electronics, CNTs are being explored for their potential in field-effect transistors [31,32], touch panels [33], and field emitters [34,35] due to their high conductivity and electron mobility. In energy-related applications, CNTs have demonstrated significant promise in supercapacitors [36,37], hydrogen storage [38,39], and lithium-ion batteries [40,41], where their high surface area and electrical properties improve energy density and charge capacity. In the biomedical field, CNTs are being researched for biosensors/bioelectrodes [42,43] and drug delivery systems [44], taking advantage of their biocompatibility and structural versatility. Additionally, through-silicon-via interposers [45] and CNT-based dry adhesives [46,47] are being developed for use in advanced manufacturing and semiconductor technologies.

ML has also proven to be highly beneficial across numerous facets of research and development of CNTs, given the nature of the involvement of a vast array of intricate and multifaceted parameters. As a special one-dimensional molecule with a unique tubular structure, controlling the atomic structures, characterizing the distinct chirality, and establishing the relationship between properties, structures, and device performance for CNTs are significantly more challenging. The complexity of CNT synthesis, which includes factors such as catalyst composition/structure, temperature, synthesis ambient, carbon feedstock, growth enhancers, and reaction conditions, presents additional significant challenges. ML can address them by identifying patterns and optimizing processes that would be difficult or time-consuming to achieve through traditional experimentation methods. Moreover, ML has facilitated the interpretation of large datasets from CNT characterizations, enabling researchers to uncover subtle correlations and predict properties with greater accuracy. This ability to manage and analyze complex datasets has proven indispensable in advancing CNT applications, where precise control over structural properties can lead to innovations in fields ranging from electronics to materials science. Consequently, the role of ML plays in CNT research continues to grow, offering the potential to unlock new possibilities and drive further progress in this cutting-edge area of nanotechnology.

This short review briefly highlights the significant contributions of the ML approach to CNT synthesis, characterizations, and applications, demonstrating how ML has substantially advanced CNT research. Finally, the review concludes with a perspective, offering insights into potential future developments and directions for deeper integrating ML into CNT research to drive the field forward.

## 2. Machine Learning-Assisted CNT Synthesis

**Figure 2:**
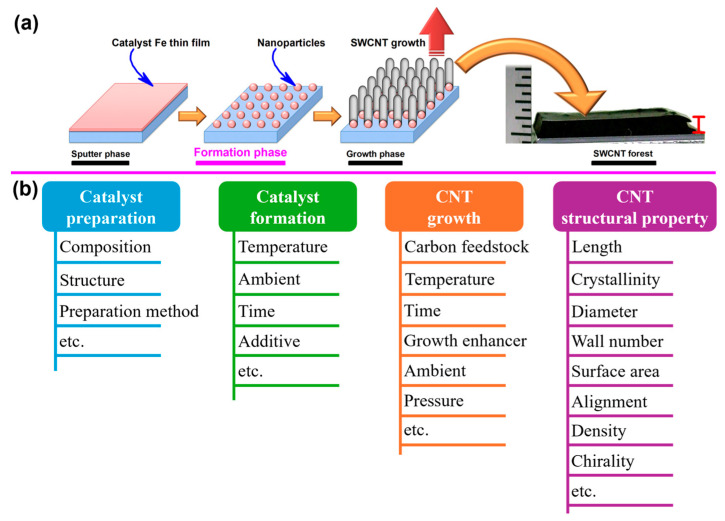
(**a**) A typical CNT CVD synthesis process. Reprinted with permission from Reference [48]. (**b**) Complex multivariable parameters in CNT synthesis process.

Synthesis of CNTs involves complex multivariable parameters. A typical CVD synthesis process of CNTs includes catalyst preparation, catalyst nanoparticle formation, and CNT growth, targeting the desired CNT structural properties (Figure 2a) [48]. As shown in Figure 2b, all these processes are highly sensitive to a vast range of parameters including, but not limited to, catalyst type (composition: commonly used elements such as Fe, Co, Ni, or their alloys; structure: single-layer or multilayer; preparation method: sputtering, evaporation, casting, spin coating, etc.), temperature, pressure, carbon feedstock (type and concentration), growth enhancer (type and concentration), ambient conditions (carrier gas type and concentration), time, etc. [49,50,51]. These parameters must be meticulously controlled to achieve CNTs with desired structural properties [52], typically including length [53,54], crystallinity [55,56], diameter [57,58], wall number [59,60,61], surface area [62,63], alignment [64,65], density [66,67], etc. Traditionally, optimizing these parameters has relied heavily on trial-and-error approaches, which are time-consuming and labor-intensive, and may not fully capture the complex interactions between these variables. ML has emerged as a powerful tool to address these challenges, offering data-driven strategies for efficiently optimizing CNT synthesis parameters and even enabling the development of autonomous synthesis systems.

### 2.1. Data-Driven Optimization of CNT Synthesis

Data-driven optimization generally refers to the use of ML algorithms to analyze large datasets generated from CNT synthesis experiments and focuses on optimizing the multitude of variables involved in the production process. By identifying patterns and correlations within the dataset, ML algorithms can model the complex relationships between synthesis parameters and the resulting properties of CNTs. These models can predict how changes in synthesis parameters will affect the properties of the resulting CNTs, facilitating the identification of optimal synthesis conditions, significantly reducing the need for trial-and-error approaches, and accelerating the discovery of targeted CNTs with desired structural properties. This approach can also provide deeper insights into the underlying mechanisms governing CNT growth.

In CNT synthesis, the parameter space is vast and multidimensional, as shown in Figure 2b. These parameters may interact in nonlinear ways to influence the structural properties of CNTs. ML algorithms such as support vector machines (SVMs), random forests (RFs), XGBoost, multilayer perceptron (MLP), Bayesian optimization, and artificial neural network (ANN) are particularly effective in exploring this parameter space. They can identify complex patterns and correlations that might be missed by traditional statistical methods. These models have been previously applied in material synthesis and showed excellent performance [16,68,69,70,71,72,73]. In the field of CNT synthesis, successful applications of data-driven optimizations have also been demonstrated [15,16,29,74,75,76,77,78].

**Figure 3:**
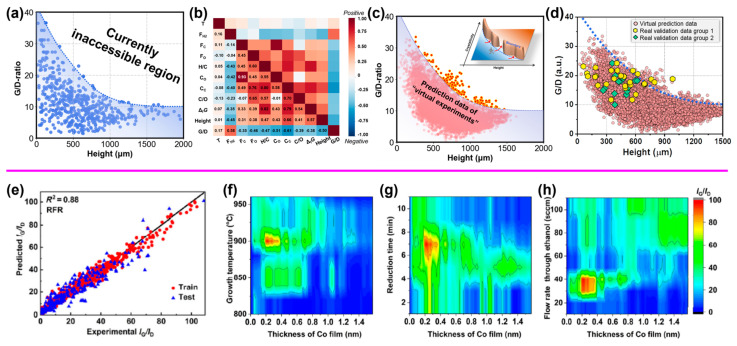
(**a**–**d**) Data-driven assisted SWCNT forest synthesis overcoming the synthetic trade-off. Reprinted with permission from Reference [16]. (**a**) Trade-off between G/D ratio and CNT height. (**b**) Heat map of Pearson correlation coefficient matrix. (**c**) Plot of virtual experiment data with prediction points beyond the boundary. (**d**) Validation results. (**e**–**h**) ML-assisted high-throughput screening for high-quality SWCNTs. Reprinted with permission from Reference [76]. (**e**) I_G_/I_D_ from experiments and predictions. Dependence of predicted I_G_/I_D_ on Co film thickness with (**f**) growth temperature, (**g**) reduction time, and (**h**) flow rate through ethanol as secondary variables.

For instance, Lin et al. demonstrated a route for addressing the synthetic trade-off between CNT forest crystallinity and growth efficiency through the assistance of data-driven optimization [16]. Specifically, synthetic trade-offs in the synthesis of SWCNT forests are very challenging as growing certain desired properties can often come at the expense of other desirable characteristics. One example is the one between crystallinity and growth efficiency, resulting in the difficulty of achieving both high crystallinity and tall forests simultaneously (Figure 3a). To address this, Lin et al. used a data-driven approach to train a machine learning regression model (XGBoost) using nine input feature descriptors with a set of 585 experimental synthesis data (Figure 3b). Subsequently, 16,000 exploratory “virtual” experiments were performed by the trained model to examine potential routes toward addressing the current crystallinity−height (growth efficiency) trade-off limitation, and possible growth conditions were predicted (Figure 3c). Finally, validation experiments showed very good agreement with the predictions, highlighting the effectiveness and accuracy of the predictive capability of the ML model, which achieved improved results in less than 50 validation tests. Importantly and fundamentally, the trained model revealed the surprising importance of the nature of the carbon feedstock, as a route for indirectly maintaining a high level of crystallinity at a high growth efficiency through the maintenance of the activity of the catalysts to overcome the trade-off. This data-driven approach represents a significant advance in complex multivariable CNT synthesis systems.

Another example is that Ji et al. used ML for high-throughput screening of the efficient growth of high-quality SWCNTs [76]. A database of 1280 experiments was built with four input parameters of the thickness of the cobalt (Co) catalyst film, growth temperature, reduction time, and carbon precursor flow rate and one output of the Raman I_G_/I_D_ ratio. ML models including linear regression (LR), random forest regression (RFR), support vector regression (SVR), and ANN were trained, and RFR was finally chosen due to its highest prediction performance (Figure 3e). The trained model was used to further explore the parameter space for the optimum growth conditions for high-quality SWCNTs with 38,016 combinations of growth parameters (Figure 3f–h). Finally, the predicted optimum growth parameters were validated to achieve a high I_G_/I_D_ value of 138 in the conventional CVD growth process. The efficient modeling, predicting, and learning ability of the combined high-throughput and ML method show great potential to speed up the controlled synthesis of SWCNTs with specific structures and properties.

In the data-driven approach, the size of the dataset plays a crucial role in determining the performance and reliability of ML models. The general thinking is that larger datasets are beneficial for training ML models because they can provide more information for the model to learn from, reducing overfitting and leading to more accurate and robust predictions. However, they also require more computational resources. Sufficient unbiased data are crucial for successful ML training and validation [79]. The appropriate dataset size depends on several factors, including the problem type, the level of complexity, the number of features, data quality, and error tolerance. This makes it a case-by-case scenario. The most widely used rule-of-thumb is that the dataset size should be at least 10 times the number of weights in the model. However, other guidelines also exist, such as requiring 50 to 1000 times the number of prediction classes or 10 to 100 times the number of features [80]. Krasnikov et al. examined the implementation of ML techniques and discussed features of the optimal dataset size and density for aerosol synthesis of SWCNTs with a complex carbon source [77]. They originally employed a dataset of 369 points comprising four inputs and three target parameters and assessed the performance of six ML methods. They showed that even a dataset of 250 points with an inhomogeneous distribution of input parameters is sufficient to reach an acceptable performance of the ANN model, wherein the error is most likely to arise from experimental inaccuracy and hidden uncontrolled variables. Therefore, careful consideration of dataset quality and input feature selection, rather than just dataset size, seems to be more important.

In summary, the use of ML in the data-driven optimization of CNT synthesis parameters not only reduces the time and resources required for CNT synthesis experimentations but also enhances the reproducibility of results. By providing a deeper understanding of the synthesis process, ML enables researchers to design experiments more strategically, leading to the discovery of new synthesis pathways and the production of CNTs with tailored properties.

### 2.2. Autonomous Synthesis of CNTs

Autonomous synthesis represents a paradigm shift in materials science, where ML and automation technologies are integrated to enable fully automated production/synthesis processes. In the context of CNT synthesis, this involves the development of systems that can autonomously adjust synthesis parameters in real time based on continuous feedback from ML algorithms, ensuring the production of CNTs with desired, precise, and consistent properties. This concept goes beyond traditional methods that rely on manual adjustments and trial-and-error experimentation. Instead, autonomous synthesis systems can dynamically adjust synthesis parameters on the fly to optimize the quality and yield of CNTs, significantly improving efficiency and reproducibility.

**Figure 4:**
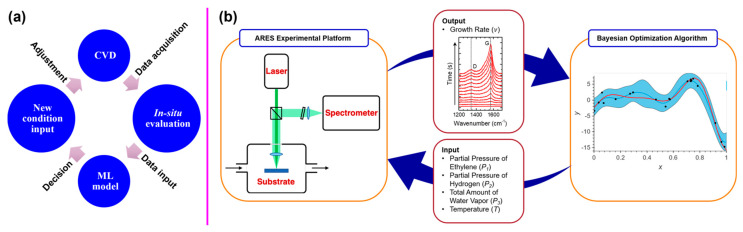
(**a**) A conceptual diagram of a closed-loop autonomous synthesis system. (**b**) A closed-loop autonomous synthesis system with adaptive rapid experimentation and in situ spectroscopy (ARES) and Bayesian optimization ML algorithm. Reprinted with permission from Reference [83].

Due to the multivariable nature of CNT synthesis, a closed-loop autonomous synthesis system is uniquely suitable for optimizing the synthesis process continuously and autonomously by integrating real-time data monitoring, ML algorithms, and automated process controls. As conceptually diagrammed in Figure 4a, a simple system generally starts with a CVD reactor to perform the synthesis process and collect the growth parameters. The system also needs to be equipped with in situ monitoring and evaluation units, such as Raman spectroscopy, thermogravimetric analysis (TGA), and mass spectrometry, to provide real-time monitoring of the process. Then, all these data are sent into an ML control unit to analyze the data and predict the optimal synthesis conditions. Finally, the decisions of new synthesis conditions are set and fed back into the CVD process.

Autonomous synthesis of CNTs has been successfully demonstrated in several experimental setups [81,82,83,84]. For instance, a proof of concept was exemplified by Maruyama et al. with an adaptive rapid experimentation and in situ spectroscopy (ARES) system (Figure 4b). This system was developed to increase the experimentation rate by 100-fold, to 100 runs per day, with results analyzed in situ and in real time via Raman spectroscopy. This system was used to synthesize CNTs on the surface of micropillars, and linear regression modeling was used to map regions of selectivity toward SWCNT and multiwall CNT (MWCNT) growth in the complex parameter space of 534 CVD experiments [81]. Recently, the same group demonstrated a closed-loop system using Bayesian optimization (BO) as an efficient and robust ML algorithm integrated with the ARES setup (Figure 4b). The CNT growth rates were extracted from the ARES and fed into the BO algorithm. The algorithm then generated a new set of conditions, run by ARES, and the new output data were sent to the BO planner to update the existing dataset and plan a new experiment. This closed-loop system significantly improved the predictive power, showed good consistency performance, exploited a complex parameter space, and achieved a 5-fold faster experimentation speed than before [83].

More recently, Zhang’s group introduced an AI-driven platform, Carbon Copilot (CARCO), which integrates transformer-based language models tailored for carbon materials, robotic CVD, and data-driven ML models. Using CARCO, a catalyst discovery was demonstrated by predicting a superior Titanium–Platinum bimetallic catalyst for high-density horizontally aligned CNT (HACNT) array synthesis, validated through over 500 experiments. With the assistance of millions of virtual experiments, an unprecedented 56.25% precision in synthesizing HACNT arrays with predetermined densities was achieved within just 43 days [84]. This work exemplifies a great advance towards the integration of AI/ML with human expertise to overcome the limitations of traditional experimental approaches.

In summary, despite some challenges, the implementation of autonomous synthesis systems could revolutionize CNT research by drastically reducing the time and cost associated with traditional synthesis methods. This approach not only enhances the scalability of CNT production but also allows for the precise control of CNT properties, which is crucial for their integration into commercial applications such as electronics, composites, and biomedical devices.

## 3. Machine Learning-Assisted CNT Characterization

The most essential question in materials science including CNT research is to establish the relation of the microstructures with synthesis conditions and physical and chemical properties. Materials characterization is performed to extract information about the structure, including imaging in real space and spectroscopy in energy space. By establishing the relationship between structure and properties, characterization is essential to understand growth mechanisms and design microstructures for desired properties.

For CNTs, structural information extracted from characterization includes purity, morphology, diameter, length, conductivity type, distribution of chirality, and so on. Transmission electron microscopy (TEM) was the key for the discovery of CNTs [85], and for the precise characterization of individual CNTs, including the chiral indices [86]. On the other hand, Raman spectroscopy has been widely used for quick statistical analysis of CNT samples, including purity, distribution of diameter, and chirality [87,88].

In conventional materials characterizations, images and spectra are acquired and analyzed manually one by one. It is challenging to reach the optimum acquisition parameters because of the trade-offs in temporal resolution, spatial resolution, field of view, data volume, acquisition speed, stability, irradiation damage, and signal-to-noise ratio (SNR). For CNTs, from images in real space, diameters and lengths are measured. From diffraction patterns in reciprocal space, crystalline structures could be analyzed by measuring the distances and angles of diffraction spots. And from spectra, peak positions are compared with existing spectra in databases to determine the substances. The conventional analysis process is time-consuming and biased by human factors.

In recent years, ML has been applied to materials characterizations to automate the analysis process and to recognize the hidden patterns in data [89,90,91,92,93,94]. It has been demonstrated that ML can enhance efficiency, increase accuracy, and most importantly, handle large datasets through high-throughput analysis. In this section, ML-assisted CNT characterization will be introduced with examples of rapid identification of Raman spectroscopy and automated chirality determination from TEM Images.

### 3.1. Machine Learning for Rapid Identification of Raman Spectroscopy

Raman spectroscopy is based on the inelastic scattering of photons with phonons. It is widely used to analyze vibrational modes and to identify molecular structures with the fingerprints in the position, width, and shape of the peaks. Raman spectroscopy has been one of the most important methods for the characterization of CNTs [88]. The purity and quality of CNTs could be extracted from the ratio of G and D bands. In addition, diameter and chirality distribution could be obtained from the radial breathing modes (RBMs) [87,95]. One of the challenges for Raman spectroscopy is the low SNR, since the inelastic Raman scattering cross-section is much smaller than the cross-section for elastic Rayleigh scattering. In recent years, ML algorithms have been applied for Raman spectroscopy-based classification and recognition [19,76,96,97].

**Figure 5:**
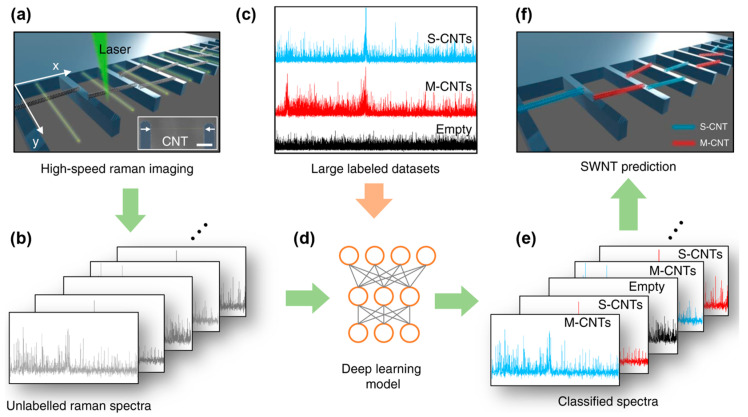
Deep learning-assisted rapid identification of Raman spectra from suspended CNTs. (**a**) Raman imaging. (**b**) Data collection. (**c**) Database labeling. (**d**) CNN deep learning model. (**e**) Training and classification. (**f**) CNT identification. Reprinted with permission from Reference [96].

For example, Zhang et al. utilized deep learning to automate and accelerate the analysis of Raman spectra for identifying structural features of suspended CNTs, including position, quantity, and metallicity (Figure 5a–f) [96]. Their model was built on a CNN with four convolutional layers, a softmax layer, and a cross-entropy layer for defining the loss function. The database consisted of 62,130 Raman spectra of suspended CNTs, categorized as semiconducting CNTs (S-CNTs), metallic CNTs (M-CNTs), and empty trenches (no CNTs). The CNN was trained on this dataset and validated on a separate set of 48,887 spectra. After training, softmax thresholding was applied to refine classification, reduce false positives, and improve accuracy. The model achieved 90% classification accuracy for spectra with the SNR as low as 0.9, with accuracy increasing to 98% for higher SNR values. The accuracy improved with longer integration times (>30 ms) and higher laser power, peaking at 99% at 1 mW laser power. This deep learning-assisted rapid identification method for CNT structures via Raman spectroscopy can be integrated into industrial production lines for real-time monitoring of CNT growth.

In another example, Kajendirarajah et al. demonstrated a rapid method for diagnosing and analyzing tip-enhanced Raman spectroscopy (TERS) mappings of individual CNTs using deep learning neural networks [97]. In this study, TERS maps were obtained from SWCNTs, where the high spatial resolution and spectral selectivity of TERS were used to distinguish between semiconductive and metallic SWCNTs, identify bundles, and locate defect areas. Two multi-purpose ANNs were developed. ANN Model 1 classified the Raman spectra from TERS experiments as either background or CNT. ANN Model 2 efficiently filtered the three vibrational modes of CNTs and accurately identified their presence in the spectra. Together, ANN Models 1 and 2 generated TERS maps with enhanced contrast and provided clear identification of the vibrational modes. The combined approach also facilitated the enhanced visualization of CNT defect regions. The deep learning ANNs achieved 98% accuracy with ANN Model 1 and 96% accuracy with ANN Model 2. This method was able to diagnose unseen raw TERS hyperspectral data in just 4 to 6 h for a spectral cube containing 8000–10,000 spectra with 1600 points each.

In summary, the utilization of ML combined with Raman spectroscopy has enabled the fast and accurate identification and classification of CNTs, resulting in a powerful and comprehensive analysis tool.

### 3.2. Machine Learning for Automated Chirality Determination from TEM Images

TEM is a key instrument for characterizing nanomaterials such as CNTs. It combines multiple functions, ranging from imaging with atomic resolution, chemical analysis of individual atoms, and mapping of local physical properties to fabrication with nanometer or even atomic precision.

For CNTs, it is quite a challenge to operate a modern TEM to precisely characterize the atomic structures because of the small atomic mass, small diameter, and complex circular geometry. It takes years to train a professional microscopist to master a variety of skills, including high-resolution TEM (HRTEM) imaging, nano-beam electron diffraction for chiral indices, STEM imaging, and 4D-STEM for probing local structures and properties.

**Figure 6:**
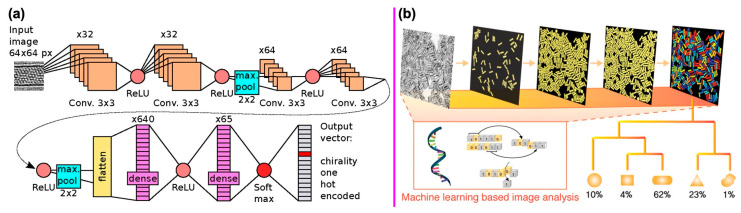
(**a**) CNN architecture for determining CNT chiral indices from HRTEM images. Reprinted with permission from Reference [101]. (**b**) Statistical characterization of nanoparticle morphologies through ML-assisted TEM image analysis. Reprinted with permission from Reference [102].

In recent years, ML methods have been applied to electron microscopy, ranging from data modeling and data analysis to atomic fabrication [27,98,99,100,101,102]. For example, Lin et al. quantified the fluctuations in catalyst carbon content by using an automated, atomic-scale structural analysis of the time-resolved ETEM images. Such fluctuations in the composition of catalysts were correlated with the SWCNT growth rate [28]. Förster et al. developed a deep learning approach for determining the chiral indices of CNTs from HRTEM images (Figure 6a) [101]. Since the chiral indices entirely determine the atomic structures and electronic and optical properties, it is critical to identify the chiral indices of individual CNTs and more important for the statistical distribution of chirality in a CNT sample. Electron diffraction is the most precise method to determine CNTs’ chiral indices; however, individual CNTs as long as 100 nm are required. In this work, Förster decided to use HRTEM images to determine the chirality of CNTs, since sub-angstrom resolution is achievable for modern Cs-corrected TEMs.

Due to the lack of enough high-quality experimental images of CNTs, a database was constructed by high-throughput atomistic structural simulation and followed by TEM simulations to generate HRTEM images. To increase the diversity of the data and generality of the model, variability was included, such as position, orientation, magnification, defocus, aberration, noise, and so on. In total, all possible 261 chiral indices for the CNTs with a diameter of 0.48 nm to 2.30 nm were considered. And for each chirality, 5000 images were simulated to general a database of 1.3 million images. A CNN based on LeNet-5 was used to model and classify simulated TEM images to identify the chiral indices. To enhance the modeling precision, two CNNs were used for the diameter and chirality, respectively. The first CNN could determine the diameter with an accuracy of 99%. And an overall accuracy of 90.5% was found for the second CNN in classifying chiral indices. The CNN-based chirality classification system was evaluated by using experimental HRTEM images. When images of sufficiently high quality were used, 71% of the results were consistent for classification conducted manually and automatically.

In this pioneering work, ML has demonstrated enhanced efficiency to identify the chiral indices, which typically takes 15–30 min to analyze one picture manually. And the model showed high robustness for the nanotubes with defects. This method opens the door for high-throughput analysis of HRTEM images to gain insight into the distribution of chirality to understand the growth mechanism of CNTs.

As is well known, the structures of CNTs are predominantly determined by the characteristics of the catalyst. Therefore, analyzing catalyst nanoparticles is essential to understanding the CNT growth mechanisms. However, due to their dynamic nature and nanoscale size, quantitatively studying nanoparticle morphologies and conducting statistical analyses on them is extremely challenging. Recently, Lee et al. developed a mass-throughput method for analyzing nanoparticle morphologies by applying a genetic algorithm to an image analysis technique (Figure 6b) [102]. This approach enabled the analysis of over 150,000 nanoparticles with a high precision of 99.75% and a low false discovery rate of 0.25%. The study also introduced clustering techniques to group nanoparticles with similar morphological shapes for extensive statistical analysis. It was determined that analyzing at least 1500 nanoparticles is required to represent the total nanoparticle population at a 95% credible interval. Additionally, the number of TEM images and the average number of nanoparticles per image should be considered to ensure a representative sample. The statistical distribution of polydisperse nanoparticles was also found to be critical in accurately estimating their optical properties.

In summary, combining ML with TEM techniques not only enhances the efficiency and accuracy of CNT analysis but also enables precise characterization of catalyst nanoparticle morphology. This integration provides a powerful tool to significantly advance the field of CNT research.

## 4. Machine Learning-Assisted CNT Applications

Besides the application in CNT synthesis, ML also plays a crucial role in enhancing the CNT applications across various fields to improve their integration into devices and materials and predict their performance. This section explores how ML aids CNT applications in electronics and sensors, composite materials, and the biomedical field.

### 4.1. CNT Applications in Electronics and Sensors

CNTs possess exceptional electrical properties, such as high conductivity, ballistic transport, and excellent current-carrying capacity, making them ideal for electronic devices and sensors [31]. However, integrating CNTs into practical devices requires precise control over their properties, processing, and placement, which can be challenging due to their nanoscale dimensions and variability in structural properties. ML can help overcome the challenges by providing data-driven insights and optimization strategies [103,104,105,106,107,108,109].

**Figure 7:**
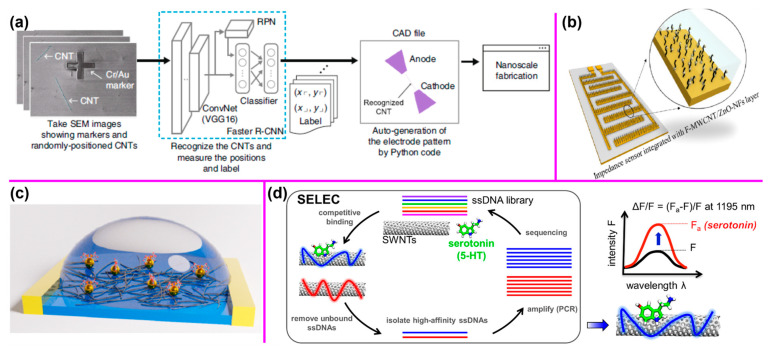
(**a**) AI-assisted framework for fabricating nanocantilevers using a neural network to recognize CNTs. Reprinted with permission from Reference [105]. (**b**) NH_4_^+^-selective impedance sensors fabricated by embedding F-MWCNT/ZnO-NF active layers on interdigitated arrays. Reprinted with permission from Reference [106]. (**c**) Schematic diagram for the composition of sensing material and liquid-gated CNT FET. Reprinted with permission from Reference [107]. (**d**) ML-assisted approach to identify DNA sequences in DNA−SWCNT conjugates with high serotonin response. Reprinted with permission from Reference [109].

For example, Tadokoro et al. developed an AI-assisted method that significantly enhanced the efficiency of fabricating CNT-based nanocantilevers (Figure 7a) compared to traditional methods like CVD and dielectrophoresis [105]. These conventional methods typically involve the manual and time-consuming placement of additional electrodes and careful observation of individually grown CNTs. In their approach, a deep neural network was trained to recognize randomly positioned single CNTs, measure their locations, and determine the precise point where an electrode should be clamped to form the nanocantilever. This AI-driven system drastically improved the recognition and measurement process, reducing the time from 12 h with manual processing to just 2 s.

Another example is that Aliyana et al. utilized an ML approach to accurately correlate the impedance variations in zinc oxide/MWCNT nanocomposite (F-MWCNT/ZnO-NFs) to NH_4_^+^ ion concentrations [106]. The proposed NH_4_^+^ sensor along with the decision-making ML model can identify and operate at specific operating frequencies to continuously collect the most relevant information from a system (Figure 7b). In addition, Bian et al. utilized ML techniques (LR, RF) to create a calibration method for Hg^2+^ sensors based on CNT field-effect transistor (FET) devices (Figure 7c) to solve sensor response saturation [107]. Such applications of ML techniques to investigate which features in the FET signal maximally correlate with concentration changes provide valuable insight into the CNT sensing mechanism and assist in the rational design of future nanosensors. Furthermore, Fan et al. proposed an efficient framework for optimizing the design of CNT FET through the integration of device physics, ML, and multi-objective optimization to expedite the early-stage development of advanced transistors [108]. Moreover, Kelich et al. trained ML models (CNN, SVN) to predict DNA sequences with strong optical responses to the neurotransmitter serotonin (Figure 7d). They discovered five DNA-SWCNT sensors with a higher fluorescence intensity response than those using only a manual screening method [109].

In summary, these studies collectively highlight the transformative potential of ML approaches in advancing the development of CNT applications in electronics and sensors. These examples underscore the potential of ML to not only solve specific technical challenges but also to revolutionize the entire process of CNT-based device development. By automating complex analyses, optimizing sensor design, and providing rapid, data-driven insights, ML approaches are poised to play a pivotal role in accelerating the innovation and deployment of CNT technologies in various electronic and sensing applications.

### 4.2. CNT Applications in Composite Materials

CNTs are widely used in composite materials to enhance their mechanical, thermal, and electrical properties [45]. However, achieving uniform dispersion and proper alignment of CNTs within the composite matrix is challenging due to the strong van der Waals forces between the nanotubes and their tendency to agglomerate. ML provides powerful tools to optimize these processes and predict the resulting composite properties, thereby enhancing the performance of CNT-based composites across various applications [110,111,112,113,114,115,116,117,118,119,120].

**Figure 8:**
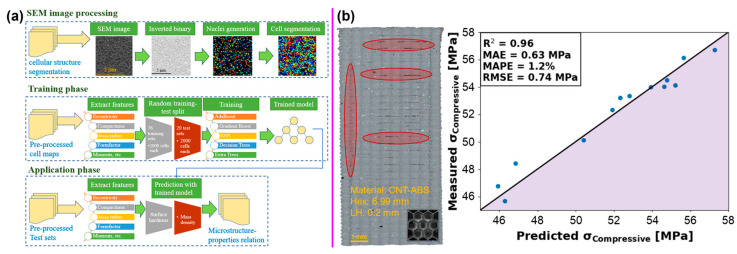
(**a**) Schematic of the proposed processing framework pipeline for AlSi10Mg nanocomposites’ microstructure–property linkages. Reprinted with permission from Reference [110]. (**b**) Optical microscopy cross-sections of CNT-reinforced ABS honeycomb composite and measured versus predicted ultimate compressive strength. Reprinted with permission from Reference [111].

For instance, Yu et al. utilized ML techniques to explore the structure–property linkages of CNT-reinforced AlSi10Mg nanocomposites [110]. The proposed processing framework pipeline starts with SEM image processing of cellular microstructural features, followed by training of ML models (AdaBoost, gradient tree boosting, K-nearest neighbors, decision tree, and extra trees regressors), and property prediction (Figure 8a). The developed models demonstrated satisfactory performance, with the extra tree regression model predicting hardness with a 2.47% error and the decision tree regression model predicting relative mass density with a 1.42% error. This framework can be applied to process optimization and mechanical property manipulation for designing new alloys or composites.

In another example, Ranaiefar et al. investigated structures and mechanical property predictions of CNT-reinforced acrylonitrile butadiene styrene (ABS) composites fabricated by 3D printing using an ML approach [111]. Multiple regression algorithms were evaluated, including ridge regressor, linear regression, k-neighbors regressor, gradient boosting regressor, random forest regressor, extra trees regressor, decision tree regressor, and lasso regression. The predictive classification and regression supervised ML models supported the experimental results with 0.92 accuracy and a 0.96 coefficient of determination (Figure 8b). This approach utilizing ML techniques can help inform and guide the design of 3D-printed structures for targeted performance.

Furthermore, Jalal et al. demonstrated a concept of ‘Big Data’ analytics in composite structure with focusing on functionally graded CNT-reinforced composites (FG-CNTRCs) using a mesh-free method and an optimized neural network (ONN) approach to study the effect of structural parameters on vibrational frequence [112]. They built big data containing 15,625 entries with six parameters and developed an optimized ONN model for predictive modeling. The ONN model demonstrated computation speeds thousands of times faster than the mesh-free method while keeping a simulation error as low as 1%, suggesting it is an efficient and reliable approach for handling big data in the optimization and design of composites.

Moreover, different ML techniques have been utilized to study various CNT-based composites for property improvements either in combination with experimental investigations, such as CNT-reinforced cementitious composites [113,114,115] and CNT-reinforced polymeric composites [116,117,118,119], or alongside computer simulations [120].

In summary, ML is revolutionizing the development and application of CNT-based composite materials by providing powerful tools for optimizing fabrication processes, predicting composite properties, and guiding the design of composites with tailored performance characteristics. By leveraging large datasets and advanced ML algorithms, researchers can overcome the challenges associated with CNT dispersion and alignment, enabling the production of high-performance composites for a wide range of applications. As ML technologies continue to evolve, their integration with composite material research and manufacturing processes will likely lead to even greater advancements in this field.

### 4.3. CNT Applications in the Biomedical Field

CNTs also possess unique properties, such as high surface area, biocompatibility, and the ability to penetrate cell membranes, making them ideal for various biomedical applications [42,121]. ML enhances these applications by providing tools for optimizing the design of CNT functionalization, predicting their interactions with biological molecules, enabling high-throughput selection of targeted DNA sequences, accelerating the discovery of effective biomolecular constructs, and even predicting their genotoxicity [122,123,124,125,126].

**Figure 9:**
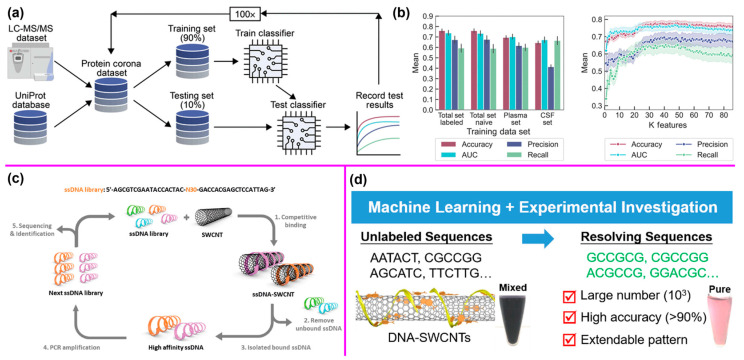
(**a**) RFC workflow and (**b**) classifier performance on different biofluid training datasets and varied protein feature inputs. Reprinted with permission from Reference [122]. (**c**) Selection of high-affinity ssDNA sequences on SWCNT surfaces. Reprinted with permission from Reference [123]. (**d**) ML-assisted systematic search of DNA sequences. Reprinted with permission from Reference [124].

For instance, Ouassil et al. developed a random forest classifier (RFC, Figure 9a) to investigate the relationship between a protein’s amino acid sequence and a protein’s binding propensity to SWCNTs [122]. The classifier aimed to predict which protein–SWCNT interactions to expect in biological environments and to predict high-affinity protein binders to SWCNTs, along with protein features associated with such binding affinity to improve the process of protein–nanoparticle construct design (Figure 9b). The model was validated with a new set of proteins by performing quantitative protein adsorption experiments. The ML classifier can serve as a useful tool for understanding how protein sequences influence nanotube binding.

Additionally, the use of single-stranded DNA (ssDNA) as a wrapping surfactant for SWCNTs is a promising approach to functionalize and modify the SWCNT surface, enabling precise control over SWCNT alignment. This approach has shown great potential in biomedical applications, including drug delivery, gene therapy, and biosensors. Lee et al. employed high-throughput systematic selection of high-affinity ssDNA sequences using ML models (RF, MLP, CNN, etc.) from a vast random library (Figure 9c). The model accurately distinguished high-affinity ssDNA sequences and provided predictive capabilities for binding affinity, supporting the design of tailored DNA-SWCNT constructs. The stability of ssDNA conformations on SWCNTs were validated by molecular dynamics simulations [123].

Furthermore, the prerequisite of utilizing DNA in sequence-dependent applications, such as biomedical sensors, is to search specific sequences, which represents a significant challenge due to the countless possible sequence combinations. Lin et al. demonstrated an ML-assisted experimental method for systematically searching DNA sequences based on sequence-dependent recognition between DNA and SWCNTs (Figure 9d). Compared with empirical search methods, their approach significantly improved both the number of resolvable sequences and the success rate of finding them, from ~10^2^ to ~10^3^ and from ~10% to >90%, respectively [124]. Moreover, ML approaches have also been successfully used to investigate CNT genotoxicity prediction [125] and discover molecular recognition based on SWCNT corona phases [126].

In summary, ML techniques enhance CNT applications in the biomedical field by enabling precise and targeted functionalization of CNTs, improving their biocompatibility, and optimizing their interaction with biological systems. Additionally, ML-driven approaches provide powerful predictive tools to accelerate the discovery and development of CNT-based biomedical constructs, expanding the possibilities for innovative therapies and diagnostics.

## 5. Conclusions and Perspective

This review demonstrates the transformative impact of ML on CNT research, covering areas such as the optimization of growth conditions, automation of structure characterization, and application development. ML algorithms have proven highly effective in modeling the complex relationships between synthesis parameters and the resulting structures and properties of CNTs by identifying patterns and correlations within datasets. As a result, improvements in the height of CNT forests and the quality of CNT thin films have been achieved. Additionally, the integration of autonomous synthesis systems has further enhanced the CNT production, reducing reliance on manual trial-and-error processes.

ML has been widely adopted for materials characterization and has started to revolutionize the CNT field by automating traditionally manual tasks. Notable successful applications include determination of the chiral indices through HRTEM images and rapid identification of suspended CNTs using Raman spectroscopy, both of which utilized deep learning for automation. ML has demonstrated enhanced efficiency to extract information to automate the analysis process, markedly improving the speed and accuracy of data analysis, facilitating high-throughput studies and more consistent results.

Moreover, ML has been accelerating the innovation and deployment of CNT-based applications in electronics and sensors, through automating complex analyses, optimizing sensor designs, and providing rapid, data-driven insights. It has also provided valuable guidance to design composites with desired properties by optimizing fabrication processes and predicting composite characteristics. In the biomedical field, ML is playing a crucial role in optimizing CNTs’ functionalization and predicting their interactions with biological molecules, furthering CNT applications in drug delivery, biosensors, and diagnostics.

Despite the significant progress, the integration of ML into CNT research is still in its early stages and holds immense potential for exponential growth in the near future. Up to now, application examples of ML in CNT characterizations are still very limited. A search using the key words “machine learning” and “carbon nanotube” in the “Web of Science Core Collection” yielded only 131 publications, with the number of publications increasing in a linear manner from 2 in 2015 to >30 in 2024.

A critical issue is the selection of an appropriate ML model for specific data types and problems. Many ML models, such as linear regression (LR), SVM, XGBoost, MLP, and Bayesian optimization, have been developed based on smart algorithms. LR is a basic model that assumes a linear relationship between input parameters and the target variable. SVM aims to find a hyperplane in a high-dimensional space that best fits the data and can model nonlinear relationships using kernel functions. XGBoost is an optimized gradient boosting algorithm that builds an ensemble of decision trees, with each tree focusing on correcting the errors of the previous ones. MLP is a type of neural network that learns nonlinear relationships through multiple layers. Bayesian optimization is a probabilistic model that optimizes by building a surrogate model (e.g., a Gaussian process) and uses it to guide exploration and exploitation. Up to now, model selection has been based primarily on performance metrics such as the coefficient of determination (R^2^) for training and test datasets. It is expected that future models incorporating physical insights and expert knowledge of growth, characterization, and applications will be constructed to be more effective in addressing the problems in materials science and CNT research.

One of the main limitations is the lack of organized data, which are indispensable for training complex ML models. Currently, there is no centralized database for CNT research that aggregates experimental data from various groups. Most advances have been driven by individual groups accumulating years of experimental data. The hunger for data is a common challenge in data-driven science. Typically, a scale of tens of thousands of data points is needed for high-accuracy deep learning modeling. However, conducting experiments is time-consuming, as CNT growth can take hours, while capturing TEM images and obtaining Raman spectra typically take minutes. Collaboration within the CNT research community, among experimentalists, theorists, and data scientists, will be the key to constructing shared CNT databases and establishing protocols for data storage, sharing, usage, and importantly protecting the precious data. Another approach to data collection is through large-scale, high-throughput calculations, though computational power remains a limiting factor. Furthermore, there is a gap between theoretical and experimental studies regarding system size and timescales. For example, a typical simulation investigates a few hundred atoms by first-principles calculation over nanoseconds, while real-world reactions involve a large number of atoms on the order of 10^23^ over hours. Moreover, although the current main problem is the availability of datasets, attention should also be given to potential bias in datasets during the data collection process. For example, most catalysts used for CNT growth are transitional metals, and this imbalance could hinder the discovery of new catalyst systems. A possible solution is to integrate and collaborate with standardized databases when CNT-specific databases are lacking. If ML models are general enough, they could be applied to CNTs as a subset of materials science. Thus, more practical applications of ML will enable a deeper understanding of CNT data patterns.

As a subset of ML, generative AI has recently gained significant attention because it not only learns from data but also generates new content, such as text, audio, and images, based on the training data. In recent years, natural language processing (NLP) models like GPT-4 have demonstrated the ability to generate human-like text and engage in conversations. Although generative AI is starting to be applied to scientific research, including materials science, its use in CNT research remains limited. However, its potential is immense and should not be overlooked. Generative AI models, such as generative adversarial networks (GANs) and variational autoencoders (VAEs), could be highly useful in future CNT research for tasks such as optimizing synthesis pathways, predicting novel CNT structures, and enabling property-driven material discovery. The integration of generative AI into CNT research has the potential to further accelerate innovation by automating more creative aspects of material design and discovery. As this area continues to evolve, we anticipate that generative AI will play a pivotal role alongside ML, opening new frontiers in CNT research.

Looking ahead, another important direction will be the development of integrated AI systems that combine growth optimization, structural characterization, and property measurements into a single closed-loop framework. By leveraging ML techniques, these systems could continuously optimize the entire CNT research process, significantly accelerating discovery and innovation. As human researchers increasingly focus on designing AI systems and making critical scientific decisions, this integrated AI-driven approach is expected to drive revolutionary breakthroughs in CNT growth mechanisms, the discovery of novel structures, and the development of unprecedented applications.

## Data Availability

Not applicable.
